# Sex-Specific Models to Predict Insulin Secretion and Sensitivity in Subjects with Overweight and Obesity

**DOI:** 10.3390/ijms24076130

**Published:** 2023-03-24

**Authors:** Myriam Beaudry, Simon Bissonnette, Valérie Lamantia, Marie Devaux, May Faraj

**Affiliations:** 1 Faculty of Medicine, Université de Montréal, Montréal, QC H3C 3J7, Canada; 2 Institut de Recherches Cliniques de Montréal (IRCM), Montréal, QC H2W 1R7, Canada; 3 Montréal Diabetes Research Center (MDRC), Montréal, QC H2X 0A9, Canada

**Keywords:** diabetes, prediabetes, insulin resistance, Botnia-clamp

## Abstract

Sex-specific differences exist in insulin secretion (ISec) and sensitivity (IS) in humans. However, current fasting indices used to estimate them, such as HOMA and QUICKI, are not sex-specific. We aimed to develop sex-specific models to improve the prediction of ISec and IS by fasting measures in adults with overweight/obesity. A post hoc analysis was conducted on baseline data of two clinical trials completed between 2010 and 2020 (37 men and 61 postmenopausal women, 45–73 years, BMI > 25 kg/m^2^, without chronic disease). Glucose-induced insulin or C-peptide secretions and IS were measured using gold-standard Botnia-clamps, which is a 1 h intravenous glucose tolerance test followed by a 3 h hyperinsulinemic–euglycemic clamp. Stepwise regression analysis using anthropometric and fasting plasma glucose, insulin, and lipoprotein-related measures was used to predict ISec and IS. First-phase, second-phase and total glucose-induced ISec were predicted by a combination of fasting plasma insulin and apoB without or with plasma glucose, triglyceride, and waist circumference in women (R^2^ = 0.58–0.69), and by plasma insulin and glucose without or with BMI and cholesterol in men (R^2^ = 0.41–0.83). Plasma C-peptide, alone in men or followed by glucose in women, predicted C-peptide secretion. IS was predicted by plasma insulin and waist circumference, followed by HDL-C in women (R^2^ = 0.57) or by glucose in men (R^2^ = 0.67). The sex-specific models agreed with the Botnia-clamp measurements of ISec and IS more than with HOMA or QUICKI. Sex-specific models incorporating anthropometric and lipoprotein-related parameters allowed better prediction of ISec and IS in subjects with overweight or obesity than current indices that rely on glucose and insulin alone.

## 1. Introduction

Anomalies in insulin secretion (ISec) and insulin sensitivity (IS) are well-known risk factors for the development of type 2 diabetes (T2D) [1]. A positive feedback loop exists between insulin resistance (IR) in peripheral tissue and ISec by pancreatic β-cells that, in time, favors β-cell exhaustion, reduced ISec, hyperglycemia, and progression to T2D [1]. A lower IS combined with insufficient ISec, measured as a lower disposition index, was reported to be an independent predictor of the conversion of prediabetes to T2D across many ethnic groups and races [2].

Many techniques have been developed to assess glucose-induced ISec and/or IS in humans using various infusions of glucose and insulin. These include the hyperinsulinemia–euglycemia (HIEG) clamp, the hyperglycemia clamp, the intravenous glucose tolerance test (IVGTT), the minimal model, the continuous infusion of insulin with model assessment (CIGMA), and, lately, the Botnia-clamp (IVGTT followed by HIEG clamp) [3,4,5]. While considered “gold-standard”, these techniques are complex, invasive, and intensive in respect to labor, time, and cost, which limits their application in large-scale population studies. Accordingly, simple indices derived from fasting plasma insulin and glucose have been developed to predict ISec and IS.

The Homeostatic Model Assessment is a computer-generated model that was developed in 1985 to estimate β-cell function, ISec (HOMA-β), and insulin resistance (HOMA-IR) from pairs of fasting plasma glucose and insulin [3]. It considers that, at fasting steady-state, the balance between hepatic glucose output and ISec is maintained by a feedback loop between the liver and β-cells [3]. The formulas for HOMA-β and HOMA-IR assume that normal-weight healthy subjects aged <35 years have 100% β-cell function and a HOMA-IR of one [3,4]. An updated nonlinear computer model, HOMA2, was later described that considers variations in peripheral and hepatic glucose resistance [6]. The curve for ISec was changed to be able to assess ISec when glycemia is >10 mmol/l, and the model included an estimation of proinsulin secretion to allow the use of different insulin assays [6]. Altogether, these changes were considered to provide a better estimation of β-cell function and IS than originally described in 1985 [6].

The quantitative insulin sensitivity check index (QUICKI) is another widely used model for IS, which is considered identical to HOMA-IR except that QUICKI uses a log transform of the glucose and insulin product [7]. Fasting plasma non-esterified fatty acids (NEFA) were later incorporated in the model (revised QUICKI) to account for the role of adipose tissue dysfunction and fat metabolism in IR [8]. HOMA-IR, HOMA-β [3], QUICKI [7], and revised QUICKI [8] were validated against measures of IS and ISec using the HIEG clamp, hyperglycemia clamps, minimal model, IVGTT, and/or CIGMA among healthy subjects with normal fasting glucose, adults living with obesity, T2D, and/or other insulin-resistant conditions [4,9,10].

However, there are well-documented sex-specific differences in IS and ISec in the regulation of glucose and insulin homeostasis in humans [11]. Specifically, using gold-standard Botnia-clamps, we reported that nondiabetic women with overweight and obesity have higher IS and lower ISec than age- and BMI-matched men with similar fasting and IVGTT-induced plasma glucose [12]. Thus, the balance between ISec and IS to maintain steady-state or glucose-stimulated plasma glucose concentrations is differently regulated in men and women. Using identical mathematical models with fasting glucose and insulin alone may not yield the best estimation of IS and ISec in both sexes.

Here, we optimized on the availability of scarce gold-standard measures of glucose-induced ISec and IS collected using Botnia-clamps that were conducted in 98 nondiabetic subjects with overweight or obesity recruited through two clinical trials by our team. We tested whether the inclusion of anthropometric and fasting-plasma lipoprotein-related parameters improves the prediction of ISec and IS beyond that predicted by insulin and glucose alone in this population.

## 2. Results

Fasting baseline characteristics and indices of insulin secretion and sensitivity during the Botnia-clamp of the 61 women and 37 men are presented in Table 1. Men had higher weight, lean body mass, and central adiposity (higher android fat, android-to-gynoid fat ratio, waist circumference, and waist-to-hip ratio). Notably, however, while the absolute value of waist circumference was higher in men, 93% of the women had abdominal obesity compared to 81% of men (defined as waist circumference ≥88 cm in women and ≥102 cm in men) [13]. Men also had lower fasting plasma total cholesterol (TC), LDL-C and HDL-C, and NEFA, and higher fasting plasma triglyceride (TG).

As previously published [12] and presented in Table 1, despite similar fasting plasma glucose and area under the glucose curve during IVGTT (AUC_IVGTT_) in this population, men had higher ISec in the fasting state (plasma insulin, HOMA-β_insulin_, and HOMA2-β_insulin_) and during the IVGTT (first-phase GIIS_IVGTT_, second-phase GIIS_IVGTT_, and total GIIS_IVGTT_ and first-phase and total C-peptide secretions). On the other hand, women had higher IS assessed at fasting (lower HOMA-IR and HOMA2-IR, and higher HOMA2-S (%) and QUICKI) and during the HIEG clamp (higher GIR_clamp_). Moreover, as previously reported [12], second-phase GIIS_IVGTT_ represented more than 75% of the total GIIS_IVGTT_ in normoglycemic men and women.

### 2.1. Sex-Specific Models to Predict Glucose-Induced Insulin Secretion (GIIS) Measured by the IVGTT

**Figure 1:**
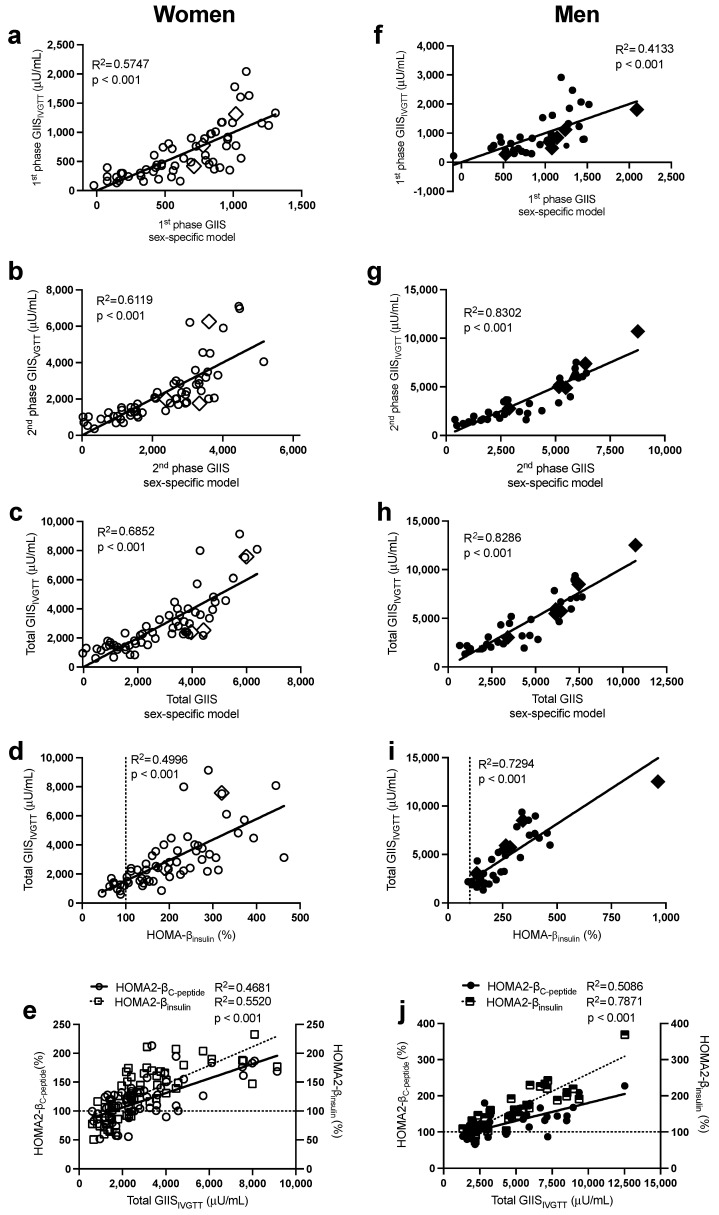
Pearson correlations between the sex-specific models, with their corresponding data for first-phase GIIS_IVGTT_ in women (**a**) and men (**f**), second-phase GIIS_IVGTT_ in women (**b**) and men (**g**), and total GIIS_IVGTT_ in women (**c**) and men (**h**), between HOMA-β_insulin_ with total GIIS_IVGTT_ in women (**d**) and men (**i**), and between total GIIS_IVGTT_ with HOMA2-β_insulin_ and HOMA2-β_C-peptide_ in women (**e**) and men (**j**). Women are in open circles (N = 61, those with BMI > 40 kg/m^2^ are in open diamonds) and men are in closed circles (N = 37, those with BMI > 40 kg/m^2^ are in closed diamonds).

**Figure 2:**
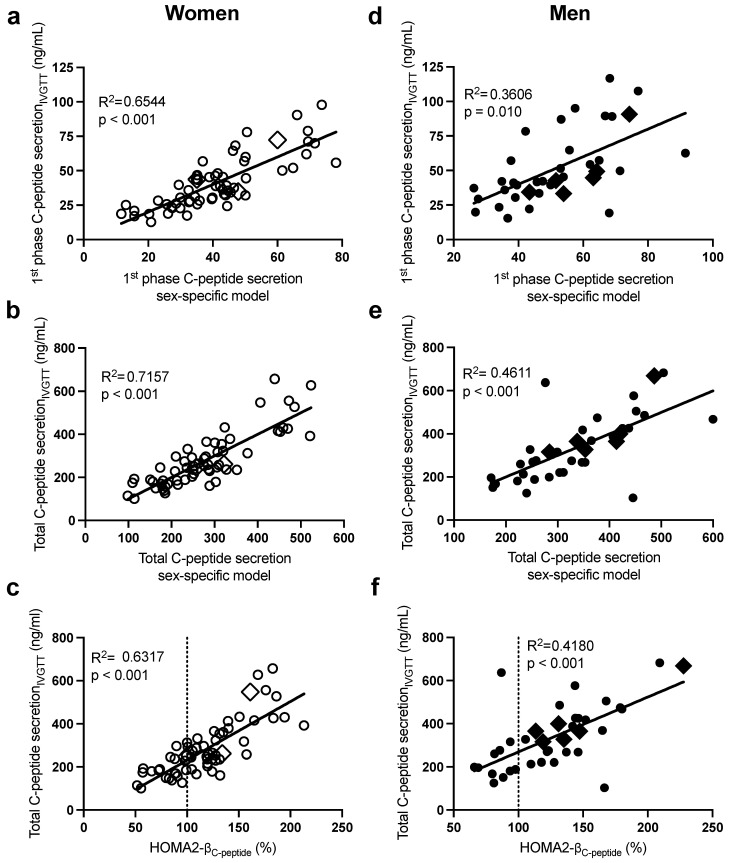
Pearson correlations between the sex-specific models, with their corresponding data for first-phase C-peptide secretion_IVGTT_ in women (**a**) and men (**d**) and total C-peptide secretion_IVGTT_ in women (**b**) and men (**e**), and between HOMA2-β_C-peptide_ with total C-peptide secretion_IVGTT_ in women (**c**) and men (**f**). Women are in open circles (N = 61, those with BMI > 40 kg/m^2^ are in open diamonds) and men are in closed circles (N = 37, those with BMI > 40 kg/m^2^ are in closed diamonds).

As previously published in subjects with overweight and obesity [12,14] and presented here, there was a large intersubject variability in first-phase, second-phase, and total GIIS_IVGTT_ (Figure 1) and first-phase and total glucose-induced C-peptide secretion_IVGTT_ (Figure 2) despite normal ranges of fasting plasma glucose. Notably, subjects with morbid obesity (BMI > 40 kg/m^2^ in diamond symbols) were distributed evenly along the range of ISec in each sex.

As presented in Table 2 (and Supplementary Table S1), the stepwise forward regression analysis revealed that in women, fasting plasma insulin, glucose, then apoB predicted 58% of the intersubject variability in first-phase GIIS_IVGTT_, while insulin and apoB alone predicted 61% that of second-phase GIIS_IVGTT_. Total GIIS_IVGTT_ was primarily predicted by plasma insulin, apoB, and glucose (R^2^ = 0.63), and the incorporation of waist circumference and TG further increased the prediction power of the model to 69%. The sex-specific models allowed a better prediction of total GIIS_IVGTT_ (R^2^ = 0.69, Figure 1c) than HOMA-β_insulin_ (Figure 1d, R^2^ = 0.50) and HOMA2-β (Figure 1e), whether calculated using plasma C-peptide (HOMA2-β_C-peptide_ R^2^ = 0.47) or plasma insulin (HOMA2-β_insulin_ R^2^ = 0.55). Only fasting plasma C-peptide and glucose were retained in the regression model to predict first-phase C-peptide secretion_IVGTT_ (R^2^ = 0.65) and total C-peptide secretion_IVGTT_ (R^2^ = 0.72) (Table 2). As with total GIIS_IVGTT_, the sex-specific model to predict total C-peptide secretion_IVGTT_ (R^2^ = 0.72, Figure 2b) was superior to HOMA2- β_C-peptide_ (R^2^ = 0.63, Figure 2c), despite the fact that both models used plasma C-peptide and glucose.

Moreover, in men (Table 2), fasting plasma insulin and then glucose were the primary predictors of the intersubject variability in first-phase GIIS_IVGTT_ (R^2^ = 0.41), second-phase GIIS_IVGTT_ (R^2^ = 0.77), and total GIIS_IVGTT_ (R^2^ = 0.78). Incorporating BMI and then plasma cholesterol further increased the power of the model to predict second-phase and total GIIS_IVGTT_ to 83%. Plasma C-peptide alone predicted first-phase (R^2^ = 0.36) and total (R^2^ = 0.46) C-peptide secretion_IVGTT_, while all other independent variables including glucose were excluded (Table 2). As with women, the sex-specific models to predict total GIIS_IVGTT_ (R^2^ = 0.83, Figure 1h) were superior to HOMA-β_insulin_ (R^2^ = 0.73, Figure 1i) and HOMA2-β (Figure 1j, R^2^ = 0.51 with insulin and R^2^ = 0.79 with C-peptide). Moreover, the sex-specific models predicting total glucose-induced C-peptide secretion were better than HOMA2-β _C-peptide_ (R^2^ = 0.46 in Figure 2e vs. R^2^ = 0.42 in Figure 2f).

### 2.2. Sex-Specific Models to Predict Insulin Sensitivity (IS) Measured by the HIEG Clamp

**Figure 3:**
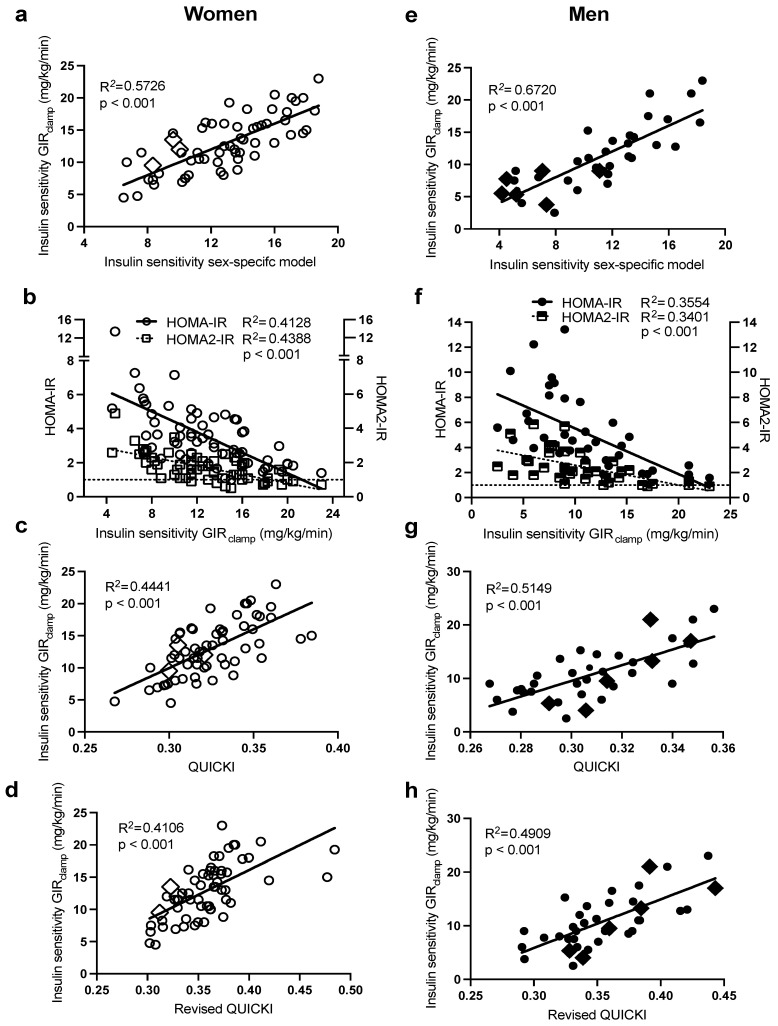
Pearson correlations between HIEG clamp measures of IS (GIR_clamp_), with the sex-specific models for IS in women (**a**) and men (**e**), HOMA-IR and HOMA2-IR in women (**b**) and men (**f**), QUICKI in women (**c**) and men (**g**), and QUICKI-FFA in women (**d**) and men (**h**). Women are in open circles (N = 61, those with BMI > 40 kg/m^2^ are in open diamonds) and men are in closed circles (N = 37, those with BMI > 40 kg/m^2^ are in closed diamonds).

As presented in Figure 3, there was also a large intersubject variability in IS in both sexes. Notably, subjects with morbid obesity (BMI > 40 kg/m^2,^ in diamond symbols) were also distributed evenly along the range of IS in each sex. Regression analysis (Table 2) revealed that plasma insulin followed by waist circumference were the primary predictors of intersubject variability in IS (GIR_clamp_) in both women (R^2^ = 0.52) and men (R^2^ = 0.57). The incorporation of plasma HDL-C in women (total R^2^ = 0.57) and glucose in men (total R^2^ = 0.67) further increased the prediction power of the sex-specific models. The sex-specific models in women (Figure 3a, R^2^ = 0.57) and men (Figure 3e R^2^ = 0.67) predicted IS (GIR_clamp_) better than HOMA-IR (R^2^: women = 0.41 and men = 0.36) and HOMA2-IR (R^2^: women = 0.44 and men = 0.34) (Figure 3b,f), QUICKI (R^2^: women = 0.44 and men = 0.51, Figure 3c,g), and revised QUICKI (R^2^: women = 0.42 and men = 0.49, Figure 3d,h).

### 2.3. Bland–Altman Plots to Assess the Agreement with the Botnia-Clamp Data

**Figure 4:**
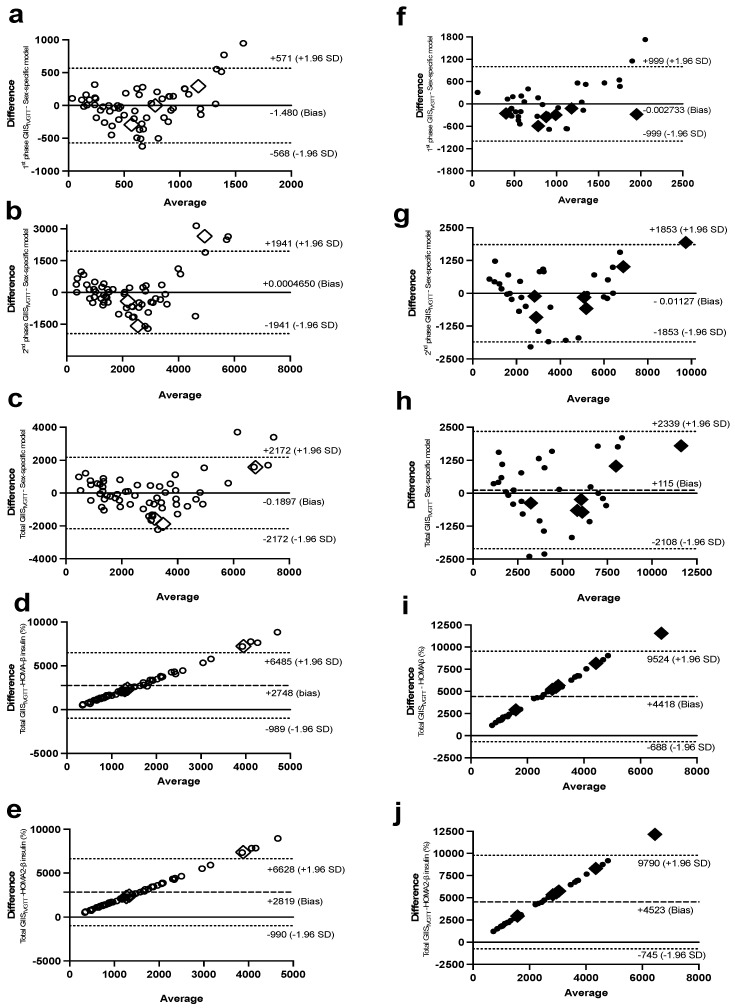
Bland–Altman plots with 95% confidence interval for limits of agreement of the sex-specific models, with their corresponding data for first-phase GIIS_IVGTT_ in women (**a**) and men (**f**), second-phase GIIS_IVGTT_ in women (**b**) and men (**g**), and total GIIS_IVGTT_ in women (**c**) and men (**h**), and between total GIIS_IVGTT_, with HOMA-β_insulin_ in women (**d**) and men (**i**), and HOMA2-β_insulin_ in women (**e**) and men (**j**). Women are in open circles (N = 61, those with BMI > 40 kg/m^2^ are in open diamonds) and men are in closed circles (N = 37, those with BMI > 40 kg/m^2^ are in closed diamonds).

**Figure 5:**
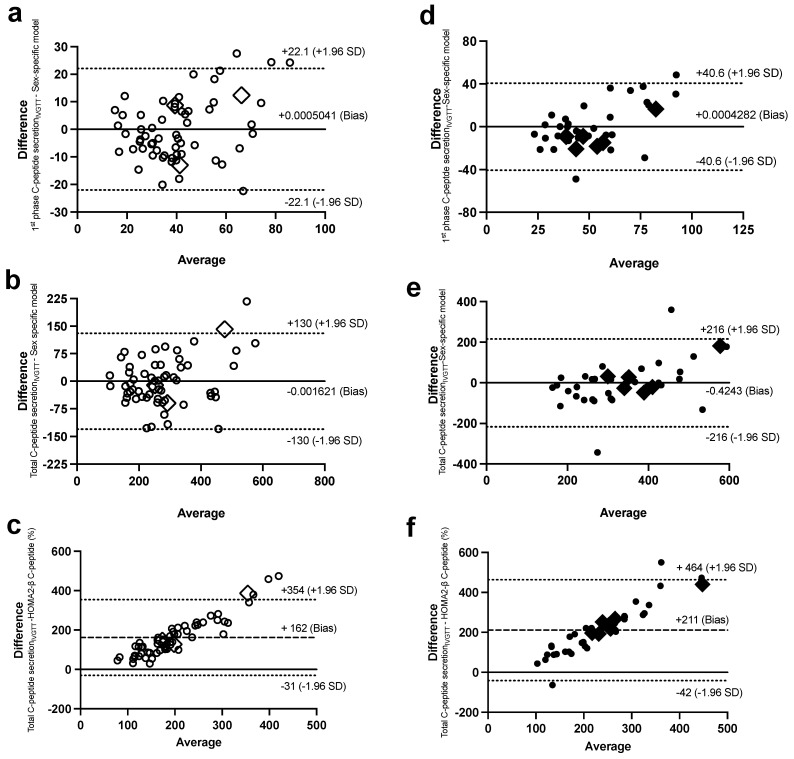
Bland–Altman plots with 95% confidence interval for limits of agreement of the sex-specific models, with their corresponding data for first-phase C-peptide secretion_IVGTT_ in women (**a**) and men (**d**) and total C-peptide secretion_IVGTT_ in women (**b**) and men (**e**), and between HOMA2-β_C-peptide_, with total C-peptide secretion_IVGTT_ in women (**c**) and men (**f**). Women are in open circles (N = 61, those with BMI > 40 kg/m^2^ are in open diamonds) and men are in closed circles (N = 37, those with BMI > 40 kg/m^2^ are in closed diamonds).

**Figure 6:**
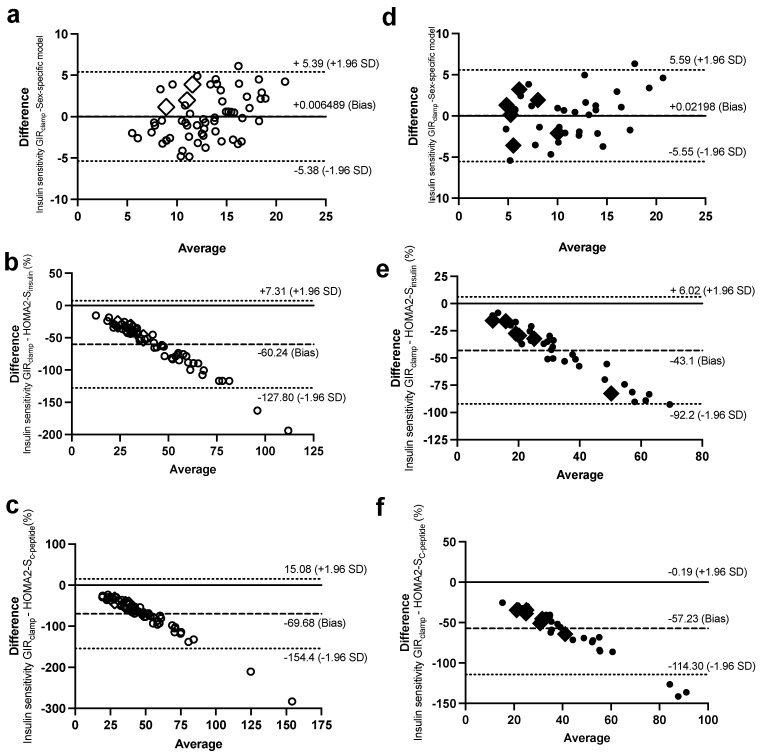
Bland–Altman plots with 95% confidence interval for limits of agreement of the IS measure during the Botnia-clamp with the sex-specific model for IS in women (**a**) and men (**d**), HOMA2-S_insulin_ in women (**b**) and men (**e**), and HOMA2-S_C-peptide_ in women (**c**) and men (**f**). Women are in open circles (N = 61, those with BMI > 40 kg/m^2^ are in open diamonds) and men are in closed circles (N = 37, those with BMI > 40 kg/m^2^ are in closed diamonds).

Bland–Altman plots were used to investigate the agreement of the sex-specific models of ISec, C-peptide secretion, and IS with their corresponding measures of the Botnia-clamp. In women, only 1–3 observations out of the total 61 observations for GIIS_IVGTT_ (Figure 4a,c), C-peptide secretion_IVGTT_ (Figure 5a,b), and IS (Figure 6a) were outside the 95% confidence interval for the limits of agreement of the two methods (Figure 4b had four observations outside the confidence interval). Similarly, in men, only 1–2 out of 38 observations for GIIS_IVGTT_ (Figure 4f–h), C-peptide secretion_IVGTT_ (Figure 5d,e), and IS (Figure 6d) were outside the 95% confidence interval for the limits of agreement of the two methods.

In comparison, there was a proportional bias when the HOMA models were used to estimate ISec, C-peptide secretion, and IS, as they did not agree equally with their corresponding Botnia-clamp measures through the range of measurements in this population. The HOMA-β and HOMA2-β models overestimated the total GIIS_IVGTT_ (Figure 4d,e for women and Figure 4i,j for men) and C-peptide secretion (Figure 5c for women and 5f for men) for subjects with lower ISec and C-peptide secretion, and underestimated them for subjects with higher ISec and C-peptide secretion.

On the contrary, the HOMA2-S models, using either insulin or C-peptide, underestimated IS for subjects with lower IS (or higher IR) and overestimated it for subjects with higher IS (Figure 4c and Figure 6b for women and Figure 6e,f for men). Data generated using the HOMA models were within the 95% limits of agreement with the Botnia measures except for total GIIS_IVGTT_ in women (5 out of 61 observations) and IS using C-peptide in men (3 out of 37 observations). Notably, however, the large biases (i.e., average difference between the Botnia-clamp measures and HOMA indices) are related to different scales used for the two measures, which was up to about 10-fold higher with the Botnia-clamp measures. Moreover, Bland–Altman plots for QUICKI and QUICKI-FFA were similar to the HOMA2-S plots but in the reverse direction.

## 3. Discussion

In this analysis, we optimized on the availability of scarce Botnia-clamp measures of glucose-induced ISec and IS in 98 middle-aged men and postmenopausal women with overweight and obesity, but no chronic disease or medication affecting metabolism, to develop sex-specific models to predict these parameters using simple fasting data. The incorporation of the anthropometric and fasting lipoprotein-related parameters in this analysis allowed better predictions and agreement with Botnia-clamp measures of ISec and IS than HOMA, HOMA2, or QUICKI models. Given the age, BMI, and sedentary lifestyle of the subjects examined in this study, they represent a population that is frequently studied for the assessment of the risk and prevention of T2D. Thus, an accurate estimation of ISec and IS is vital for risk assessment and follow-up through various interventions. As normally conducted, the validation of the sex-specific models described here is warranted if they were to be applied in different populations.

The existence of a two-phase response of ISec following an acute rise in plasma glucose was first shown by Cerasi et al. [15]. The first phase represents the fusion of a small “readily releasable pool” of granules (~50–200) that are pre-docked [16] or close to [17] the plasma membrane, leading to the quick discharge of insulin within 10 min. This phase is crucial to restoring glucose homeostasis after the rise in plasma glucose, and is the first to decline during the progressive loss of β function and T2D [18,19]. The second phase represents a “reserve pool” of storage granules and produces a substantial and prolonged ISec that is larger than the first phase [16], as also demonstrated in this study (Supplementary Figure S1a,b). In normal glucose-tolerant subjects, ISec peaks during the first phase and slowly decreases to a more sustained secretion until the plasma glucose concentration has returned to steady-state homeostasis [20]. To our knowledge, the present work is the first report of sex-specific models to predict IS and glucose-induced ISec, which is particularly scarce when assessing first-phase and second-phase ISec separately. Furthermore, prediction models of ISec from plasma C-peptide are also uncommon. However, plasma C-peptide is a better index of ISec than plasma insulin, given its longer plasma half-life compared to insulin (20–30 min for C-peptide versus 3–5 min for insulin) [21]. Plasma C-peptide is also cleared by the kidney and not the liver, which makes it less affected by hepatic and systemic IR than plasma insulin [21].

It is of no surprise that in both women and men, higher fasting plasma insulin and C-peptide were the primary predictors of first-phase, second-phase, and total glucose-induced insulin and/or C-peptide secretions, respectively. However, depending on the sex, different secondary parameters were retained to predict ISec and C-peptide secretions. Lower plasma glucose was the secondary predictor of first-phase GIIS_IVGTT_ and first-phase and total C-peptide secretions in women and of all measures of GIIS_IVGTT_ (first-phase, second-phase, and total) in men. However, it was not retained to predict C-peptide secretions in men, as only fasting plasma C-peptide was needed, which had a higher range of measurements in men than women. Moreover, plasma apoB was superior to glucose as a second predictor of second-phase and total GIIS_IVGTT_ in women, while higher plasma total cholesterol also added to the prediction of second-phase and total GIIS_IVGTT_ in men but only after the inclusion of BMI.

The influx and accumulation of apoB-lipoproteins in peripheral tissues are known to induce lipotoxicity, IR, and β-cell dysfunction [22,23,24,25]. Plasma apoB is a measure of the number of plasma apoB-lipoproteins (mostly in the form of LDL), and higher plasma apoB is associated with a smaller particle size, which facilitates particle uptake and tissue dysfunction [12,14,24,25]. Previous work from our team reported the superiority of plasma apoB compared to lipids in its association with second-phase and total insulin and C-peptide secretion measured during IVGTT [12,14,24,25]. Interestingly, when plasma TG was retained to predict total GIIS_IVGTT_ in women, it was in the reverse direction, which may be a reflection of the inverse regulation of plasma TG by plasma insulin. Notably, the inclusion of healthy subjects with overweight and obesity but without chronic disease or high cardiovascular risk (i.e. Framingham risk factor > 20%) may have introduced a selection bias. This is because age has a higher impact on the calculation of Framingham risk factor in men than women, and it is more likely to include women aged 45–74 years with higher plasma lipid-related parameters than age-matched men.

Higher plasma insulin followed by a larger waist circumference were the primary predictors of lower IS in both women and men, while lower plasma glucose further added to the prediction power of the model in men only. Plasma glucose was replaced by plasma HDL-C in women. HDL is reported to have beneficial effects on cholesterol homeostasis and to suppress inducible nitric-oxide synthase and fatty-acid synthase in β-cells [26]. As plasma HDL-C was higher in women, it may have favored a greater impact. It should be noted that, in simple correlation analysis, fasting plasma glucose is negatively associated with IS, but to a lesser extent in women (r = −0.28) than in men (r = −0.52) (*p* < 0.05). However, its contribution to IS in women was eliminated once other variables that had a higher correlation to IS were selected in the regression model (i.e., fasting plasma insulin, waist circumference, and then fasting plasma HDL-C).

The sex-specific models of ISec and IS were compared to the commonly used HOMA and HOMA2. The original HOMA models described in 1985 by Matthews et al. were calibrated with insulin assays used in the 1970s and were later reported to underestimate IS and, therefore, overestimate ISec when compared with newer insulin assays [4]. The use of HOMA models was still advised for when ISec and IS were compared between populations and when their longitudinal changes, using the same insulin assays, were followed [4]. The use of the newer nonlinear HOMA2 models [6] was later recommended for the assessment of absolute IR and β-cell function, as they better accounted for hepatic and peripheral IR, renal glucose loss, and increases in ISec with hyperglycemia, and they were calibrated using newer assays and were extended to allow the use of C-peptide [4]. Their availability online also allowed their update using newer assays [4]. Nevertheless, in our cohort, separating the two sexes and accounting for the influence of anthropometric and fasting lipoprotein-related parameters in addition to insulin and glucose allowed a better prediction of IS and ISec derived from the Botnia-clamps compared to HOMA, HOMA2, or QUICKI models. Furthermore, the Bland–Altman plots demonstrated a clear proportional bias generated by all HOMA models (i.e., the limits of agreement depend on the actual measurement in each population). These models underestimated insulin and C-peptide secretions as well as IS in subjects with higher diabetes risk (with lower IS and higher GIIS), but overestimated them in subjects with lower diabetes risk (with higher IS and lower GIIS). This bias may reduce the validity of the HOMA models, including HOMA2 models, to estimate absolute IR and β-cell function in a similar population with overweight and obesity.

It should be underscored that other indices derived from oral glucose-tolerance tests (OGTT), or standardized liquid mixed-meal tests were also developed to predict glucose-induced ISec and IS measured by IVGTT or HIEG clamps [27,28,29], including an IS index developed by our group in postmenopausal women with obesity [27]. However, an OGTT or a meal is still needed to be administered to obtain the data needed to calculate these indices. Moreover, OGTT and meal challenges add intersubject variability in relation to intestinal glucose absorption and incretin secretion and their cumulative effects on pancreatic ISec, a variability that is bypassed when ISec and C-peptide secretion are measured by IVGTT or estimated using fasting indices. That is why we believe estimating ISec and IS measured during Botnia-clamps are best predicted by fasting measures. The effect of glucagon-like peptide 1 on ISec has been evaluated in recent studies [29], and with this measure, OGTT-derived indices are best used to predict ISec, as was conducted in that study [29].

In conclusion, this work developed sex-specific models to predict Botnia-clamp-generated measures of IS and glucose-induced ISec and C-peptide secretion using simple fasting clinical parameters in sedentary men and postmenopausal women with overweight and obesity who represent a high-risk population for the development of T2D. Separating the two sexes and incorporating anthropometric and fasting plasma lipoprotein-related parameters increased the power to predict ISec, C-peptide secretion, and IS in this population compared to the commonly used HOMA and QUICKI models, which are not sex-specific.

## 4. Methods

### 4.1. Study Population

A post hoc analysis was conducted on pooled baseline data of two registered trials conducted between 2010 and 2020 at the Institut de recherches cliniques de Montréal (IRCM) (ISRCTN14476404 and NCT04496154 [14]). The objective was to test whether including simple anthropometric and/or fasting plasma lipoprotein-related parameters improves the prediction of ISec and IS beyond that predicted by insulin and glucose alone in subjects with overweight and obesity. For both trials, subjects were recruited by newspaper advertisement and online with the following inclusion criteria: 45–74-year-old men and postmenopausal women (confirmed FSH ≥ 30 U/I) with a BMI > 20 kg/m^2^ who were nonsmokers, sedentary (less than 2 h of exercise/week), and with low/moderate alcohol consumption (<2 drinks/day). The exclusion criteria were: history of cardiovascular disease and hypertension requiring medication, diabetes (or fasting glucose > 7 mmol/L), cancer (within the last 3 years), untreated thyroid disease, kidney disease (or creatinine > 100 μmol/L), hepatic disease (or ALT or AST > 3 times normal limit), anemia (Hb < 120 g/L), blood coagulation problems, claustrophobia, current or past-3-months use of drugs affecting metabolism (hormone-replacement therapy, except thyroid hormone at stable dose, systemic corticosteroids, antipsychotic/psychoactive drugs, anticoagulant, weight loss, and adrenergic agonist), known substance abuse, exceeding the annual allowed radiation dose exposure, and all other medical or psychological conditions deemed inappropriate according to the physician. All subjects signed an informed consent before initiation of each trial, which were approved by the ethics committee of the IRCM.

Out of the 122 subjects recruited for the 2 trials, 8 subjects participated in both trials, 7 were excluded for missing data on ISec, IS, or waist circumference, 6 had a BMI ≤ 25 kg/m^2^, and 3 were outliers in all analyses (for BMI > 50 kg/m^2^, fasting insulin, or HDL-C). Thus, this analysis was conducted on 98 subjects (37 men and 61 women).

### 4.2. Anthropometric and Biochemical Parameters

Subjects were placed on a 4-week weight-stabilization period (±2 kg) before initiating both trials. Total, abdominal, and gluteofemoral fat mass were measured by dual-energy X-ray absorptiometry (DEXA). Fasting plasma lipids and apoB were measured by an automated analyzer (COBAS Integra 400, Roche Diagnostic, Laval, QC, Canada), glucose by an automated analyzer (YSI Incorporated, InterScience, Saint-Norm-la-Bretèche, France), and insulin and C-peptide by a radioimmunoassay kit (Millipore Corporation, Burlington, MA, USA) [12,14].

### 4.3. Insulin Secretion and Sensitivity

ISec and IS were assessed using a modified Botnia-clamp as previously published [12,14]. The advantage of the Botnia-clamp is that it allows the assessment of ISec independently of IS separately via 2 consecutive tests run on the same day [5]. Prior to the clamp, participants followed a 3-day high-carbohydrate diet (300 g/day for men and 225 g/day for women) to maximize glycogen stores. On the clamp day, subjects underwent a 1 h IVGTT using a bolus infusion of 20% dextrose (0.3 g glucose/kg body weight) (Supplementary Figure S1a,b). This was followed by a 3 h HIEG clamp using a primed-constant insulin infusion (75 μU/m^2^/min) while plasma glucose was maintained within fasting range (4.5–5.5 mmol/L) with 20% dextrose infusion (Supplementary Figure S1c,d). The area under the IVGTT plasma curves of insulin and C-peptide were used to calculate 1st-phase (first 10 min), 2nd-phase (last 50 min), and total (60 min) glucose-induced ISec (GIIS_IVGTT_) and C-peptide secretion_IVGTT_, respectively. Insulin sensitivity (IS) was assessed as glucose infusion rate (GIR_clamp_) during the steady-state of the HIEG clamp (last 30 min).

Fasting index HOMA-IR was calculated as [fasting glucose (mmol/L) × fasting insulin (μU/L)]/22.5 and HOMA-β_insulin_ was calculated as [20 × fasting insulin (μU/L)]/[fasting glucose (mmol/L) −3.5] [3]. The updated HOMA2 models (HOMA2-IR, HOMA2-S, and HOMA2-β) were calculated using the online sheet available at www.OCDEM.ox.ac.uk (accessed on 7 September 2021) [4], and HOMA2-β was calculated using fasting plasma insulin (HOMA2-β_insulin_) and C-peptide (HOMA2-β_C-peptide_). For HOMA2, the conversion factor for insulin was 1 μU/mL = 6 pmol/L [30] and that for plasma C-peptide was 3 ng/mL = 1 nmol/L [21]. The QUICKI IS index was calculated as 1/[log fasting insulin (μU/mL) + log fasting glucose (mg/dl)] [7] and revised QUICKI as 1/[log fasting insulin (uU/mL) + log fasting glucose (mg/dl) + log fasting NEFA (mmol/L)] [8].

### 4.4. Statistical Analysis

Data are presented as mean ± SD in Table 1. Sex differences were assessed by an unpaired t-test (2-tailed). Stepwise forward regression analysis was used to predict Botnia-clamp-derived indices of ISec and IS using BMI, waist circumference, fasting plasma glucose, insulin, total cholesterol, LDL-C, HDL-C, TG, NEFA, and apoB as independent variables in each sex separately. Fasting C-peptide was used instead of fasting insulin to predict C-peptide secretion in the regression models. Pearson correlation was used to evaluate the association between the independent variables and the dependent variables (Botnia-clamp measures of IS and ISec). As linearity failed for simple correlations with plasma insulin and TG, their data was LOG_10_ transferred before being entered in the regression models. Bland–Altman plots with the difference between Botnia-clamp measures of ISec and IS and the sex-specific models (or HOMA indices) on the *y*-axis versus the average of these measures on the *x*-axis, including 95% confidence intervals of the limits of agreement, were reported. Statistical analysis was conducted using IBM SPSS Statistics (version 26) and GraphPad Prism 9 with significance set as *p* < 0.05.

## Figures and Tables

**Table 1:** Baseline anthropometric and plasma parameters as well as fasting and Botnia-clamp measures of insulin secretion and sensitivity in the study population.

Characteristics	Women (N = 61)	Men (N = 37)	*p* Value
Age (years)	58.4 ± 5.84	56.4 ± 7.16	0.147
Systolic blood pressure (mmHg) ^1^	121 ± 16	129 ± 11	0.002
Diastolic blood pressure (mmHg) ^1^	76 ± 9	81 ± 8	0.001
Anthropometric measurements Weight (kg)	81.8 ± 12.7	101 ± 19.4	<0.001
BMI (kg/m^2^)	32.0 ± 4.38	33.3 ± 5.09	0.212
Total fat mass (kg)	37.2 ± 8.62	36.5 ± 13.3	0.778
Lean body mass (kg)	41.6 ± 4.73	61.3 ± 7.8	<0.001
Android fat mass (kg)	3.49 ± 1.0	4.27 ± 1.71	0.015
Gynoid fat mass (kg)	6.27 ± 1.40	5.04 ± 2.09	0.002
Android/gynoid (kg/kg)	0.56 ± 0.11	0.87 ± 0.19	<0.001
Waist circumference (cm)	101 ± 11.5	114 ± 14.0	<0.001
Hip circumference (cm) ^2^	113 ± 12.1	112 ± 12.7	0.773
Waist/hip ratio	0.90 ± 0.07	1.02 ± 0.06	<0.001
Fasting blood parameters Plasma glucose (mmol/L)	5.13 ± 0.57	5.22 ± 0.47	0.424
Plasma insulin (μU/mL)	15.3 ± 7.28	22.1 ± 12.2	0.003
Plasma C-peptide (ng/mL)	2.01 ± 0.84	2.36 ± 0.91	0.062
HbA_1_C (%) ^3^	5.65 ± 0.35	5.49 ± 0.50	0.103
Plasma total cholesterol (mmol/L)	5.54 ± 0.94	5.04 ± 0.95	0.013
Plasma non-HDL cholesterol (mmol/L)	4.03 ± 1.01	3.99 ± 1.00	0.831
Plasma LDL cholesterol (mmol/L)	3.36 ± 0.80	2.95 ± 0.71	0.010
Plasma HDL cholesterol (mmol/L)	1.51 ± 0.35	1.05 ± 0.20	<0.001
Plasma TG (mmol/L)	1.48 ± 0.87	2.26 ± 1.61	0.009
Plasma NEFA (mmol/L)	0.56 ± 0.19	0.41 ± 0.13	<0.001
Plasma apoB (g/L)	0.99 ± 0.26	1.04 ± 0.25	0.381
Fasting indices of insulin secretion and sensitivity			
HOMA-IR	3.65 ± 1.98	5.19 ± 3.03	0.004
HOMA-β_insulin_ (%)	200 ± 98.5	260 ± 161	0.049
HOMA2-IR	1.70 ± 0.81	2.35 ± 1.24	0.006
HOMA2-S (%)	73.1 ± 36.8	54.9 ± 28.1	0.007
HOMA2-β _insulin_ (%)	129 ± 42.9	155 ± 59.0	0.023
HOMA2-β_C-peptide_ (%)	117 ± 36.4	127 ± 38.7	0.235
QUICKI	0.32 ± 0.02	0.31 ± 0.02	0.004
Revised QUICKI	0.36 ± 0.04	0.36 ± 0.04	0.781
Botnia-clamp measures of insulin secretion and sensitivity			
1st-phase GIIS_IVGTT_ (μU/mL/10 min)	654 ± 446	942 ± 666	0.024
2nd-phase GIIS_IVGTT_ (μU/mL/50 min)	2290 ± 1589	3739 ± 2293	0.001
Total GIIS_IVGTT_ (μU/mL/60 min)	2948 ± 1975	4680 ± 2740	0.001
AUC_IVGTT_ glucose (mmol/L)	654 ± 86.2	663 ± 82.8	0.609
1st-phase C-peptide secretion_IVGTT_ (ng/mL/10 min)	41.2 ± 19.1	51.7 ± 25.9	0.037
Total C-peptide secretion_IVGTT_ (ng/mL/60 min)	279 ± 125	338 ± 150	0.049
Plasma insulin at steady state_clamp_ (μU/mL)	234 ± 72.5	261 ± 84.4	0.110
GIR_clamp_ (mg/kg/min)	12.9 ± 4.21	10.9 ± 4.96	0.047

Data are presented as mean ± SD. ^1^ n = 60 women, ^2^ n = 36 men, ^3^ n = 59 women due to missing data.

**Table 2:** Stepwise regression analysis to predict insulin secretion and sensitivity measured during the Botnia-clamp in women (n = 61) and men (N = 37).

Models	Steps	Independent Variables	Constant	Coefficient	R^2^	*p* Value
Women 1st-phase GIIS_IVGTT_			178			
	1	Log_10_ Insulin		1409	0.359	<0.001
	2	Glucose		−303	0.511	<0.001
	3	ApoB		438	0.575	0.005
2nd-phase GIIS_IVGTT_			−5458			
	1	Log_10_ Insulin		5683	0.567	<0.001
	2	ApoB		1298	0.612	0.013
Total GIIS_IVGTT_			−6240			
	1	Log_10_ Insulin		7283	0.548	<0.001
	2	ApoB		2632	0.600	<0.001
	3	Glucose		−894	0.632	0.002
	4	Waist circumference		31	0.662	0.036
	5	Log_10_ Triglycerides		−1963	0.685	0.049
1st-phase glucose-induced C-peptide secretion_IVGTT_			76.0			
	1	C−peptide		18.3	0.493	<0.001
	2	Glucose		−13.9	0.654	<0.001
Total glucose-induced C-peptide secretion_IVGTT_			319			
	1	C-peptide		129	0.650	<0.001
	2	Glucose		−58.1	0.716	<0.001
Insulin sensitivity (GIR_clamp_)			30.9			
	1	Log_10_ Insulin		−8.37	0.432	<0.001
	2	Waist circumference		−0.130	0.524	<0.001
	3	HDL-C		3.03	0.573	0.013
Men 1st-phase GIIS_IVGTT_			1369			
	1	Log_10_ Insulin		1863	0.285	<0.001
	2	Glucose		−539	0.413	0.010
2nd-phase GIIS_IVGTT_			−5934			
	1	Log_10_ Insulin		7850	0.720	<0.001
	2	Glucose		−1137	0.767	0.005
	3	BMI		104	0.802	0.007
	4	Total cholesterol		413	0.830	0.028
Total GIIS_IVGTT_			−4859			
	1	Log_10_ Insulin		9652	0.706	<0.001
	2	Glucose		−1680	0.778	<0.001
	3	BMI		108	0.804	0.018
	4	Total cholesterol		466	0.829	0.038
1st-phase glucose-induced C-peptide secretion_IVGTT_			11.6			
	1	C-peptide		17.0	0.361	<0.001
Total glucose-induced C-peptide secretion_IVGTT_			75.6			
	1	C-peptide		111	0.461	<0.001
Insulin sensitivity (GIR_clamp_)			55.9			
	1	Log_10_ Insulin		−8.43	0.434	0.001
	2	Waist circumference		−0.134	0.567	0.001
	3	Glucose		−3.63	0.672	0.003

## Data Availability

The datasets generated during and/or analyzed during the current study are not publicly available due to ethical restrictions but are available from the corresponding author upon reasonable request.

## Supplementary Materials

The following supporting information can be downloaded at: https://www.mdpi.com/article/10.3390/ijms24076130/s1.
