# Supplementary material for: Sex-Specific Models to Predict Insulin Secretion and Sensitivity in Subjects with Overweight and Obesity
Source: Int J Mol Sci. 2023 Mar 24;24(7):6130. doi: 10.3390/ijms24076130 (PMC10094028; doi:10.3390/ijms24076130)
Supplement: Supplementary file 1 [file ijms-24-06130-s001.zip › ijms-2280478-supplementary.pdf]

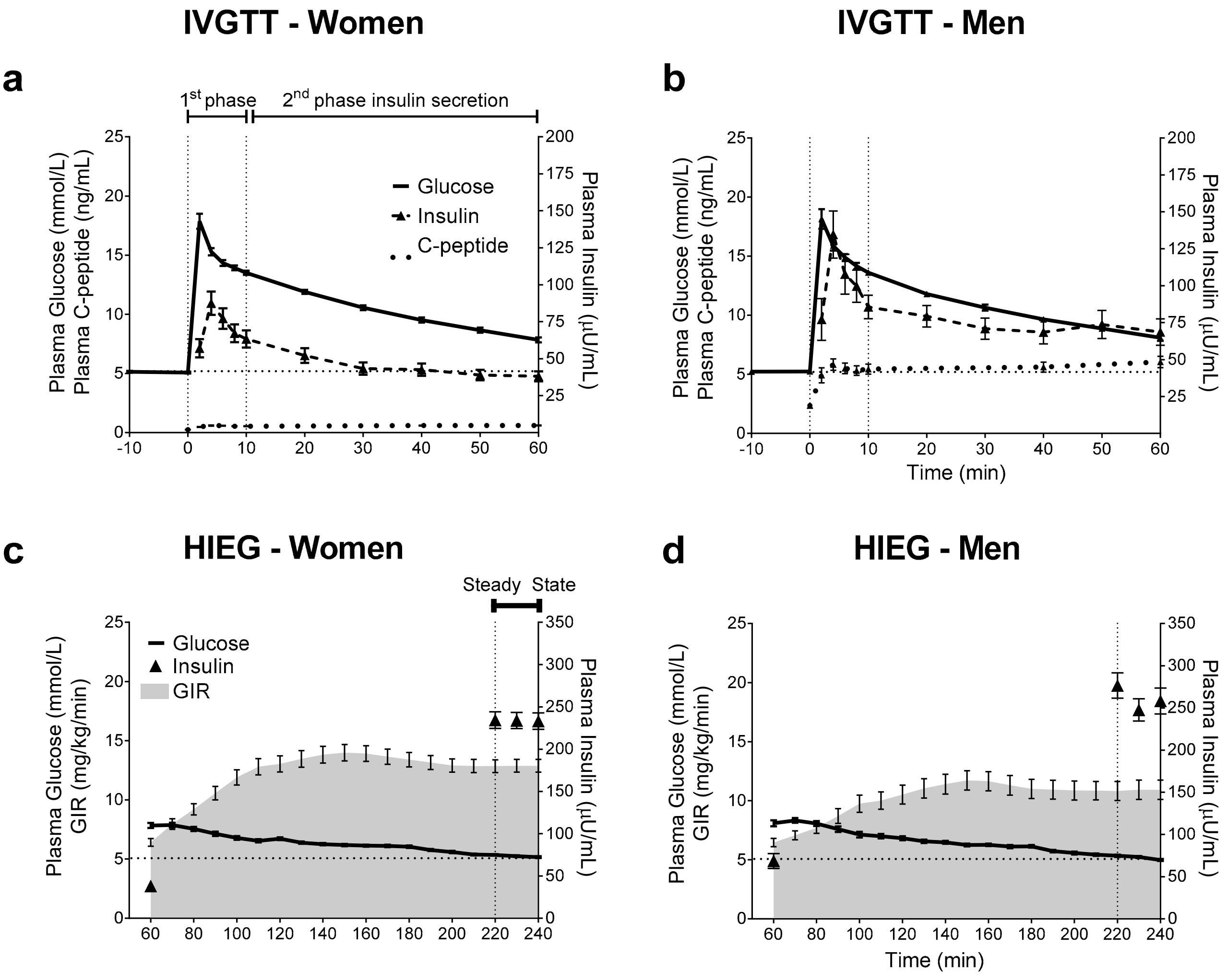

Supplemental Figure 1: Plasma glucose, insulin, and C-peptide concentrations, and glucose infusion rates (GIR) during the IVGTT in women (**a**) and men (**b**) and the hyperinsulinemic euglycemic clamp in women (**c**) and men (**d**), which as the 2 component tests of the Botnia clamps conducted in non-diabetic postmenopausal women (N = 61) and age-matched men (N = 37) with overweight and obesity.

Supplemental Table 1: Sex-specific models to predict 1st phase, 2nd phase and total GIISIVGTT, glucose-induced C-peptide secretionIVGTT and insulin sensitivity (GIRclamp) measured during the Botnia-clamps in 98 middle-aged men and postmenopausal women with overweight and obesity, but no chronic disease or medication affecting metabolism.

**Women**	
1st phase $GIIS_{IVGTT}$	= 178 + (Log_{10} Insulin x 1409) – (Glucose * 303) + (ApoB *438)
2nd phase $GIIS_{IVGTT}$	= – 5458 + (Log_{10} Insulin * 5683) + (ApoB * 1298)
Total $GIIS_{IVGTT}$	= – 6240 + (Log_{10} Insulin * 7283) + (ApoB * 2632) – (Glucose * 894) + (Waist * 31) – (Log_{10} TG * 1963)
1st phase C-peptide $secretion_{IVGTT}$	= 76 + (C-peptide * 18.3) – (Glucose * 13.9)
Total C-peptide $secretion_{IVGTT}$	= 319 + (C-peptide * 129) – (Glucose * 58.1)
Insulin sensitivity (GIR_{clamp})	= 30.9 – (Log_{10} Insulin * 8.37) – (Waist * 0.130) + (HDL-C * 3.03)
**Men**	
1st phase $GIIS_{IVGTT}$	= 1369 + (Log_{10} Insulin * 1863) – (Glucose * 539)
2nd phase $GIIS_{IVGTT}$	= – 5934 + (Log_{10} Insulin * 7850) – (Glucose * 1137) + (BMI * 104) + (TC * 413)
Total $GIIS_{IVGTT}$	= – 4859 + (Log_{10} Insulin * 9652) – (Glucose * 1680) + (BMI * 108) + (TC * 466)
1st phase C-peptide $secretion_{IVGTT}$	= 11.6 + (C-peptide * 17.0)
Total C-peptide $secretion_{IVGTT}$	= 75.6 + (C-peptide * 111)
Insulin sensitivity (GIR_{clamp})	= 55.9 – (Log_{10} Insulin * 8.43) – (Waist * 0.134) – (Glucose * 3.63)
